# Uganda’s laboratory human resource in the era of global health initiatives: experiences, constraints and opportunities—an assessment of 100 facilities

**DOI:** 10.1186/s12960-020-0454-5

**Published:** 2020-02-18

**Authors:** Suzanne N. Kiwanuka, Noel Namuhani, Martha Akulume, Simeon Kalyesubula, William Bazeyo, Angela N. Kisakye

**Affiliations:** 1 School of Public Health, Makerere University, Kampala, Uganda; 2 East African Public Health Laboratory Networking Project (EAPHLNP), Kampala, Uganda; 3 African Field Epidemiology Network, Kampala, Uganda

**Keywords:** Human resource, Laboratory sector, Uganda

## Abstract

**Introduction:**

Laboratories are vital in disease diagnosis, prevention, treatment and outbreak investigations. Although recent decades have seen rapid advancements in modernised equipment and laboratory processes, minimal investments have been made towards strengthening laboratory professionals in Africa. This workforce is characterised by insufficient numbers, skewed rural-urban distribution, inadequate qualifications, inadequate skill-mix and limited career opportunities. These factors adversely affect the performance of laboratory professionals, who are the backbone of quality services. In the era of Global Health Initiatives, this study describes the status of laboratory human resource and assesses the experiences, constrains and opportunities for strengthening them in Uganda.

**Methods:**

This paper is part of a study, which assessed laboratory capacity in 21 districts during December 2015 to January 2016. We collected data using a laboratory assessment tool adapted from the WHO and USAID assessment tool for laboratory services and supply chain (ATLAS), 2006. Of the 100 laboratories, 16 were referral laboratories (hubs). To assess human resource constraints, we conducted 100 key informant interviews with laboratory managers and in charges.

**Results:**

Across the facilities, there was an excess number of laboratory technicians at Health Center (HC) IV level by 30% and laboratory assistants were in excess by 90%. There was a shortage of laboratory technologists with only 50% of the posts filled at general hospitals. About 87.5% of hub laboratories had conducted formal onsite training compared to 51.2% of the non-hub laboratories. Less than half of HC III laboratories had conducted a formal onsite training; hospital laboratories had not conducted training on the use and maintenance of equipment. Almost all HC III laboratories had been supervised though supervision focused on HIV/AIDS. Financial resources, workload and lack of supervision were major constraints to human resource strengthening.

**Conclusion:**

Although opportunities for continuous education have emerged over the past decade, they are still threatened by inadequate staffing, skill mix and escalating workload. Moreover, excesses in staffing are more in favour of HIV, TB and malaria. The Ministry of Health needs to develop work-based staffing models to ensure adequate staff numbers and skill mix. Staffing norms need to be revised to accommodate laboratory technologists and scientists at high-level laboratories. Training needs to extend beyond HIV, TB and malaria.

## Introduction

Medical and public health laboratory services are a key constituent of a country’s health system, because they facilitate disease diagnosis, prevention, surveillance, treatment monitoring and outbreak investigations. Despite this, laboratory services and systems remain among the most neglected components of the overall health system in resource-poor countries [1]. Laboratories in resource-poor countries face a number of challenges including lack of national laboratory policies and strategic planning, insufficient numbers of trained professionals, poor laboratory infrastructures and the absence of quality management systems [2]. Over the past two decades, there have been efforts to strengthen laboratories so as to meet the requirements of several major global health programmes including the Global Fund to Fight AIDS, Tuberculosis, and Malaria (GFATM); the United States President’s Emergency Plan for AIDS Relief (PEPFAR) and the International Health Regulation [1]. These efforts have manifested as infrastructure development, diagnostic and therapeutic technology advances and advanced information systems. Despite these advances, there are persisting constraints in laboratory systems and one of these is manpower. Laboratories require manpower with specialised skills to exercise diverse functions among which are operating and maintaining equipment, managing logistics, identifying emerging public health problems and applying such information for public health action [3]. However, laboratory professionals continue to be among the most neglected cadres in health systems across sub-Saharan Africa [4]. There are often insufficient numbers, a skewed rural-urban distribution, inadequate qualifications, inadequate skill-mix, limited continuous education opportunities and limited career opportunities. Moreover, laboratory personnel often work in facilities, which are poorly equipped, and do not systematically respect safety and infection control standards. These factors adversely affect the performance of laboratory professionals, who are the backbone of quality services [5]. Furthermore, with the rapid advances in technology and emerging pandemic threats, there is need to constantly keep these professionals up to date and well equipped.

In Uganda, laboratories are essentially based in health care facilities. These laboratories are classified according to the level of health facility where they are based. These included health centres III laboratories, health centre IV laboratories, general hospital laboratories, regional referral laboratories, national referral laboratories and specialised laboratories such as Central Public Health Laboratories (CPHL), Uganda Virus Research Institute and Uganda Health Research Organisation. The laboratory services offered at each level are proportional to the complexity of medical services accessible at each level. Services are provided by pathologists, laboratory scientists, technologists, technicians and laboratory assistants.

### The laboratory system in Uganda: the hub innovation

Uganda currently has approximately 2300 laboratories. Of these, two are National Referral Hospital Laboratories (NRHL) that offer routine and specialised laboratory services and act as referral centres for lower facilities. There are 13 regional referral hospitals (RRH) in the country, which perform diagnostic tests in support of clinical services at the regional hospitals. These offer specialist and referral services within the respective regions, conduct training for health laboratory staff in collaboration with training institutions, maintain records for laboratory information and forward data to CPHL and implement laboratory National External Quality Assessment Service (NEQAS) activities in the region. They are also mandated to provide technical supervision to facilities below them. The regional referral laboratories provide the highest level of service in the region. They are headed by regional principal technologist and staffed with medical technologists, technicians, microscopists and phlebotomists. Test menus differ across facilities based on the level of facility and consist of Automated Clinical chemistry, Hematology and Leucocyte Immunophenotyping, TB diagnosis, Malaria microscopy, Urinalysis and Basic serology (VDRL, Hep. B and HIV).

At the district level, there are 64 general hospital (GH) laboratories that provide microscopy, serology, routine chemistries and haematology. There are three University Laboratories (i.e. Makerere, Mbarara and Gulu Universities)

In order to improve the efficiency and quality of service delivery, Uganda introduced the hub system where 102 hubs were set up to serve a network of 2365 facilities nationwide. The laboratory hubs have all had infrastructural improvement and received additional laboratory personnel and modern laboratory equipment to be able to conduct chemistry, hematology and CD4 as a bare minimum. The hubs are responsible for analysing samples from facilities within their catchment areas (i.e. within a radius of 30 to 40 km) and refer samples that cannot be analysed to more specialised laboratories.

The innovative hub-based National Samples and Results Transport Network (NSRTN) was initiated in 2011 to increase access to quality laboratory services by creating local networks based at hospitals with adequate laboratory capacity at the sub-district level [6]. This transport system comprises of a motorcycle rider who regularly picks samples from peripheral laboratories to deliver them to hub facilities for testing. Thereafter, test results are forwarded online to the peripheral laboratories.

By 2015, all the hub laboratories had been enrolled in the Strengthening Laboratory Management Towards Accreditation (SLMTA) programme which focuses on quality improvement of laboratories [7]. However, up until 2017, the majority (80%) of laboratories have remained at a two-star rating based on the WHO AFRO stepwise Laboratory Quality Improvement Process Towards Accreditation (SLIPTA), and only three laboratories including Mbale regional referral hospital, the chemistry and haematology laboratories in Mulago National referral hospital had been able to achieve five-star status [8].

### Strengthening human resources in the laboratory sector

**Fig. 1:**
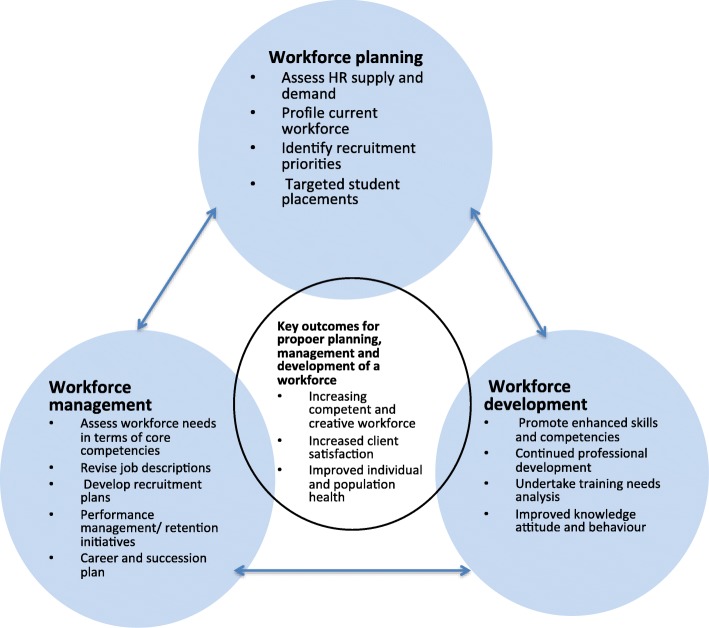
A framework for planning, managing and developing the workforce (Adapted from Bryant et al. [9])

Human resources for the laboratory sector can work competently to improve population and individual health if there are systems in place that support proper planning, management and development of the workforce (Fig. 1). Planning for the workforce involves a systematic assessment of current and future staffing requirements in terms of numbers and competencies, formulation and implementation of plans to meet those requirements [10]. In the laboratory sector, it is important that human resource managers match the workforce to the longer-term needs of the sector and maintain an ongoing review of how to make the best use of current and future workforce [11]. Retention of staff is critical for the laboratory sector since costly life-saving equipment can be rendered useless in the absence of competent personnel to operate it. When planning for the laboratory sector, it is critical to ensure that clear job descriptions are created and that pre-service training, as well as continued training plans for staff, is provided.

Uganda has 51 specialised laboratory technical schools that offer training for different qualified laboratory cadres [12]. Despite optimal production, the laboratory professionals serving public facilities in rural settings are predominantly lower cadre laboratory assistants and microscopists. Most of the higher cadre professionals such as laboratory technologists, scientists and pathologists are unwilling to serve in the public sector due to the inadequate resources, low compensation and poor career advancement opportunities within the medical laboratory profession [13]. Moreover, onsite continuous educational opportunities are often not uniformly available in the areas of professional development, quality management systems and career advancement for laboratory staff [14]. Although Uganda has made progress in strengthening human resources for health, laboratory personnel have not received due attention. This study describes the status of laboratory human resource and assesses current practices. We also conducted a bottleneck analysis to identify constrains and opportunities for strengthening human resource in the laboratory sector in the era of Global Health Initiatives (GHIs) in Uganda. Based on our findings, we propose strategies for strengthening human resources in the laboratory sector.

## Methods

### Study design and setting

This was a cross-sectional study that used mixed methods of data collection. The study was conducted in all the five different regions (Northern, Western, West Nile, central and Eastern) of Uganda. Although the National and Regional Referral Hospital laboratories in Kampala district from Central region were assessed in this study, data from these specialised laboratories are excluded from the analysis due to the uniqueness of these laboratories.

### Study population and sampling

The study respondents were 100 laboratory managers from 100 selected laboratories. This sample of 100 laboratories, with five laboratories selected randomly from 20 districts across four regions was considered representative of the whole country. The five laboratories from each district included one regional referral hospital, one general hospital, HC IV and HC III laboratories. Of these 100 laboratories, 57 were HC IIIs and 18 were HC V level laboratories. Nineteen were hospital laboratories while six were regional referral hospital laboratories. Sixteen laboratories assessed were hubs while 84 were non-hubs.

### Data collection procedures

We collected data in the months of December 2015 and January 2016. For the purpose of assessing the human resource strengthening initiatives in the laboratory sector, we collected data using a laboratory assessment tool adapted from the WHO and USAID assessment tool for laboratory services and supply chain (ATLAS), 2006. The laboratory assessment tool was used to assess the laboratory national guidelines, personnel, testing services, specimen referral, quality assurance, logistic management of laboratory supplies, inventory management, logistic management information system and transport for laboratory supplies.

A key informant interview guide was used to assess the human resource constraints affecting laboratory service delivery. This was also administered to the laboratory managers until data saturation was reached.

To assess the human resource gaps in the laboratory health sector, we interviewed laboratory managers about the staffing at the different laboratories and compared the laboratory staffing at the time of the interview with the staffing norms set at a national level.

### Data management and analysis

Quantitative data were entered in EPI Info version 7 software, cleaned and analysed using SPSS, Excel and STATA. Human resource gap analysis/staffing levels were obtained by comparing the available number of staffs for the different cadres with the MOH of staffing norms based on the level of health facilities [15] .

Qualitatively, interviews were tape-recorded, transcribed verbatim and then analysed manually using a thematic content approach. A coding framework based on the research questions was developed. The transcripts were coded and analysed, comparing and contrasting themes between different health facility levels.

## Results

### Staffing levels at laboratories in Uganda

In terms of staffing norms mostcadre did not have established standards. There, was a 30% excess in the number of laboratory technicians at the HC IV level and laboratory assistants were in excess by 90%. However, there was a shortage of laboratory technologists with only 50% of the posts filled at general hospitals and only 28% filled at regional referral hospitals. At the health centre III level, the staffing level for laboratory technicians was at 73.4% as shown below in Table 1.

**Table 1:** Staffing levels and staff mix across different levels of service delivery

Cadre	Facility level			
	HCIII = 57	HC IV = 18	Hospital = 19	RRH = 6
	Norm/filled (%)	Norm/filled (%)	Norm/filled (%)	Norm/filled (%)
Laboratory scientific officer	…-/0	…-/0	…-/9	…-/3
Laboratory technologists	…0/11	…0/2	38/*14(36.8)*	24/*10(41.7)*
Laboratory technician	57/37(64.9)	18/23(127.8)	38/27(71.1)	18/27(150.0)
Laboratory assistants	57/55(96.5)	18/*32(177.8)*	*19/47(247.4)*	*12/24(200.0)*
Laboratory attendants	…-/ 14	…-/ 2	…-/ 8	…-/ 0
Microscopists	...-/6	…-/3	…-/4	…-/0
Janitors (cleaning staff)	…-/74	…-/23	…-/29	…-/7
Total (lab technician, assistant and technologist)	114/103(90.4)	36/57(158.3)	95/88(92.6)	54/61(113.0)

Staffing norms [15]… - Staffing norm not yet set at national level

We also found that both hub and non-hub laboratories had shortages of laboratory technologists. Only 37.5% of the posts were filled for non-hub hospital laboratories, 35.7% posts were filled for hub hospital laboratories (Table 2).

**Table 2:** Staffing levels according to hub and non-hub laboratories

Cadre	Non-hub (*N* = 84)			Hub (*N* = 16)			
	HCIII = 55	HC IV = 17	Hospital = 12	HC III = 2	HCIV =1	Hospital =7	RRH = 6
	Norm/filled (%)	Norm/filled (%)	Norm/filled (%)	Norm/filled (%)	Norm/filled (%)	Norm/filled (%)	Norm/filled (%)
Scientific officer	. … -/0	…-/0	…-/9	….-/0	….-/0	….-/3	….-/3
Lab technologists	...0/11*	…0/2*	24/*9(37.5)*	…0/0	…0/0	14/*5(35.7)*	24/*10(41.7)*
Lab technician	55/*36(65.5)*	17/22(129.4)	24/*15(62.5)*	2/1(50.0)	1/1(100.0)	14/12(85.7)	18/27(150.0)
Lab assistants	55/53(96.4)	17/32(188.2)	12/34(283.3)	2/2(100.0)	1/0(0.0)	7/13(185.7)	12/24(200.0)
Lab attendants	…-/14	…-/2	…-/8	…-/0	…-/0	..-/1	…-/0
Microscopist	-/6	…-/3	…-/4	…-/0	…-/0	…-/4	…-/0

Staffing norms [15]…- Staffing norm not yet set at national level

*Laboratory technologists funded by projects

Almost all the key informants cited a number of human resource constraints. These included inadequate staff numbers, absenteeism, lack of training, lack of contracts, workload, poor management and lack of support from health facility managers and political leaders all of which constrain service delivery as demonstrated by the following quotes.

*“May be now we talk about human resources being low, since this one is a HCIV, its operating as a district hospital, all the referrals from the lower units are referred here, because the other hospital is Private not for profit (PNFP), and there is some user fee, people fear to go there, so all patients come here, yet we are few to handle that work load.”* (KI-HC IV, Oyam district).

*“…I feel, alone I am not enough in this laboratory, there could be one more staff also.”* (HCIII, Arua district)

*“Now like in the laboratory we are supposed to have a lab personnel, who is supposed to use microscopes to diagnose other diseases, things like stool examination, TB, and my concern is since he is not there, we cannot do TB tests…”* (HCIII, Terego district)

### Human resource development: training and supervision

We found that less than half (43.9%, *n* = 25) of the HC III level laboratories had conducted a formal onsite training in the past 12 months and most trainings lasted 1–5 days. However, none of the hospital laboratories had conducted training on the use and maintenance of equipment. Almost all 57 (94.7%) HC III level laboratories had been supervised and most supervision for laboratories focused on HIV/AIDS. Cold chain inspection was the least (*n* = 16, 29.6%) conducted activity at the health centre III level **(**Table 3**)**.

**Table 3:** Human resources development experiences across different levels of facilities in Uganda, 2017

Variable	Facility level			
	HCIII *n* = 57(%)	HCIV *n* = 18(%)	G. hospital *n* = 19(%)	RRH *n* = 6(%)
Trainings on *laboratory safety, TB, malaria and HIV* in the last 12 months				
Formal training on site	25(43.9)	10(55.6)	16(84.2)	6(100.0)
Formal training at national laboratory	11(19.3)	7(38.9)	9(47.4)	5(83.3)
Formal training at international laboratory	3(5.3)	0(0.0)	2(10.5)	3(50.0)
Any informal training on site	29(50.9)	11(61.1)	14(73.7)	6(100.0)
Duration of training				
1 day or less	16(64.0)	6(60.0)	10(62.5)	6(100.0)
2–5 days	06(24.0)	3(30.0)	2(12.5)	1(16.7)
1–2 weeks	03(12.0)	1(10.0)	4(24.0)	2(33.3)
Last supervisory visit				
< 3 months	45(78.9)	16(88.9)	13(68.4)	3(50.0)
3 months to < 6 months	5(8.8)	1(5.6)	3(15.1)	0(0.0)
6 months	4(7.0)	0(0.0)	2(10.5)	0(0.0)
Never	*3(5.3)*	1(5.6)	1(5.3)	0(0.0)
Institution which conducted supervision				
MOH (CPHL, NTRL, UVRI)	20(37.0)	9(52.9)	14(77.8)	6(100.0)
DHT (District Laboratory Focal Person (DLFP), District Health Officer (DHO))	37(68.5)	14(82.4)	13(72.2)	0(0.0)
Implementing partners	20(37.0)	9(52.9)	6(33.3)	0(0.0)
Focus of the supervision				
Integrated programme	46(85.2)	13(76.5)	15(83.3)	3(50.0)
Vertical programme	7(13.0)	3(17.6)	3(16.7)	3(50.0)
Do not know	1(1.9)	1(5.9)	0(0.0)	0(0.0)
Programmes supervised				
Malaria	31(57.4)	10(58.8)	11(61.1)	3(50.0)
Sexually Transmitted Inifections (STI)	21(38.9)	8(47.1)	7(38.9)	3(50.0)
HIV	*40(74.1)*	*13(76.5)*	*14(77.8)*	*4(66.7)*
Tuberculosis (TB)	35(64.8)	12(70.6)	14(77.8)	4(66.7)
Others [general lab practices, Quality Control (QC), biosafety]	22(40.7)	6(35.2)	5(27.8)	1(16.7)

About 87.5% (14) of hub laboratories had conducted a formal onsite training compared to 51.2% (43) of the non-hub laboratories. Only one hub and 10.9% of the non-hub laboratories had conducted training on equipment maintenance and none of the hub laboratories had conducted training on malaria testing procedures. All the hub laboratories had ever been supervised, while 5(6.0%) had never received any supervisory visit. The supervision largely focused on multi-programmes in both hub (68.8%) and non-hub laboratories (78.6%). However, there were gaps in the inspection of cold chain records (34.5%) and stock cards (45.2%) (Table 4).

**Table 4:** Human resources strengthening experiences in hub and non-hub laboratories, 2017

Variable (yes)	Hub *n* = 16(%)	Non-hub *n* = 84 (%)
Formal training on site	14(87.5)	*43(51.2)*
Formal training at national laboratory	8(50.0)	24(28.6)
Formal training at international laboratory	3(18.8)	5(60.0)
Any informal training on site	13(81.2)	47(56.0)
Duration of training		
1 day or less	14(87.5)	61(72.6)
2–5 days	0(0.0)	21(25.0)
1–2 weeks	2(12.5)	2(2.4)
Last supervisory visit		
Within the last month	7(43.8)	47(56.0)
≥ 1 month to < 3 months	6(37.5)	20(23.8)
≥ 3 months to < 6 months	1(6.2)	8(9.5)
≥ 6 months	2(12.5)	4(4.8)
Never	0(0.0)	*5(6.0)*
Person conducted supervision		
MOH (CPHL, NTRL, UVRI)	14(87.5)	35(41.7)
DHT (DLFP, DHO)	7(43.7)	57(67.9)
Implementing partners (Baylor, Strengthening TB and HIV&AIDS Responses in East Central Uganda Project (STAR EC), USAID RHITES-South West Project (SW project), The Maternal and Neonatal Implementation for Equitable Systems (MANIFEST), USAID, ACCESS, SUSTAIN)	4(25.0)	31(36.9)
Focus of the supervision		
Multi-programme	11(68.8)	66(78.6)
One programme	*5(31.2)*	*11(13.1)*
Don’t know	0(0.0)	2(2.4)
Programmes supervised		
Malaria	8(50.0)	47(56.0)
STI	7(43.8)	32(38.1)
HIV	10(62.5)	61(72.6)
TB	11(68.8)	54(64.3)
Others [general lablaoratory practices, QC, biosafety]	4(25.0)	30(35.7)

The inadequacy of skills and lack of training were also reported to hinder human resource performance in the laboratory sector as stated below:

*“…and there are no trainings, we are getting, there are things (equipment, procedures) brought without training us, so it’s a very big challenge. A program is just brought and you start without knowing what to do, then you end up making mistakes.”* (HCIV, Lira district)

### Bottleneck analysis of human resource strengthening in Uganda’s laboratory sector

**Fig. 2:**
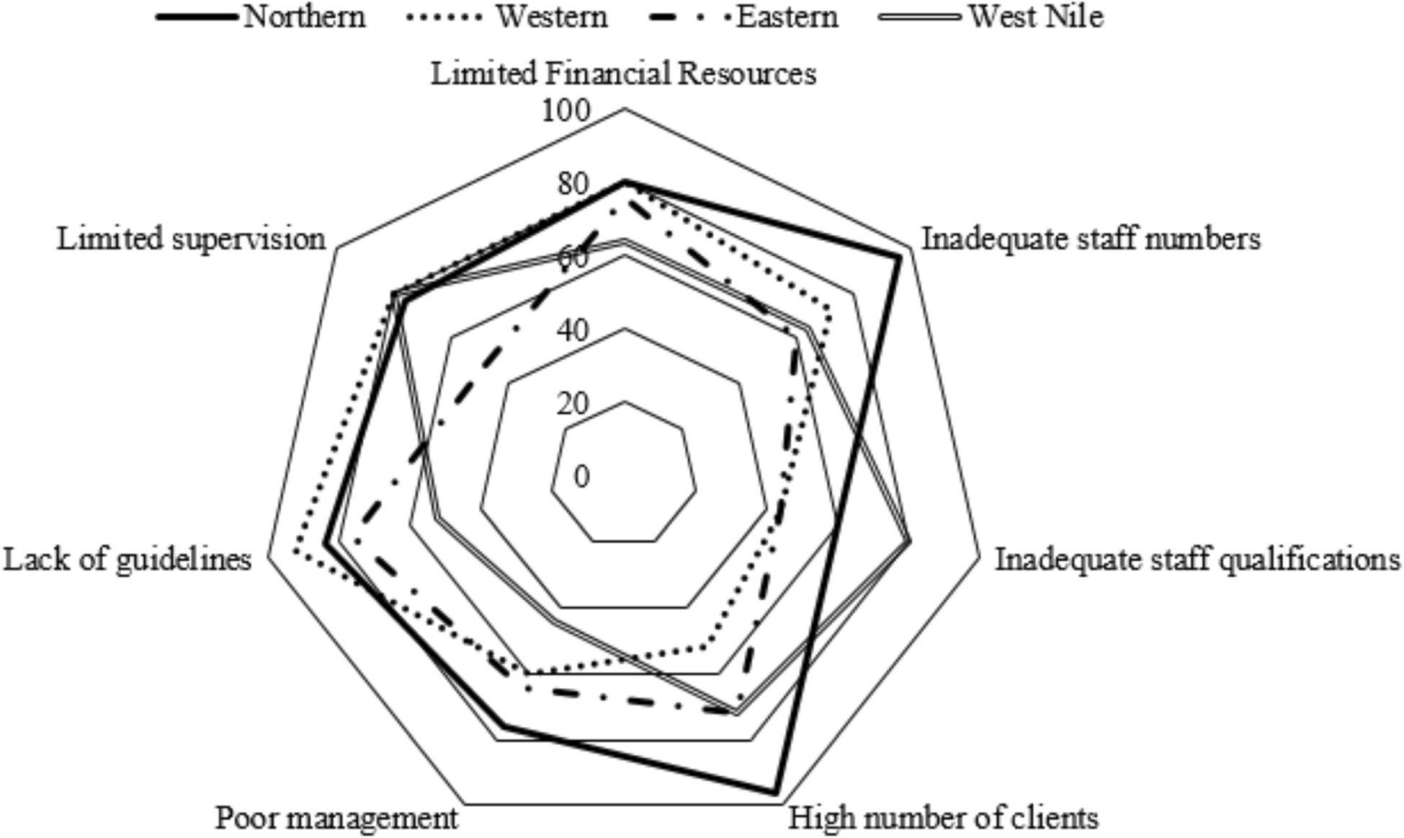
Constraints to human resource strengthening in the laboratory sector at regional level, 2017

**Fig. 3:**
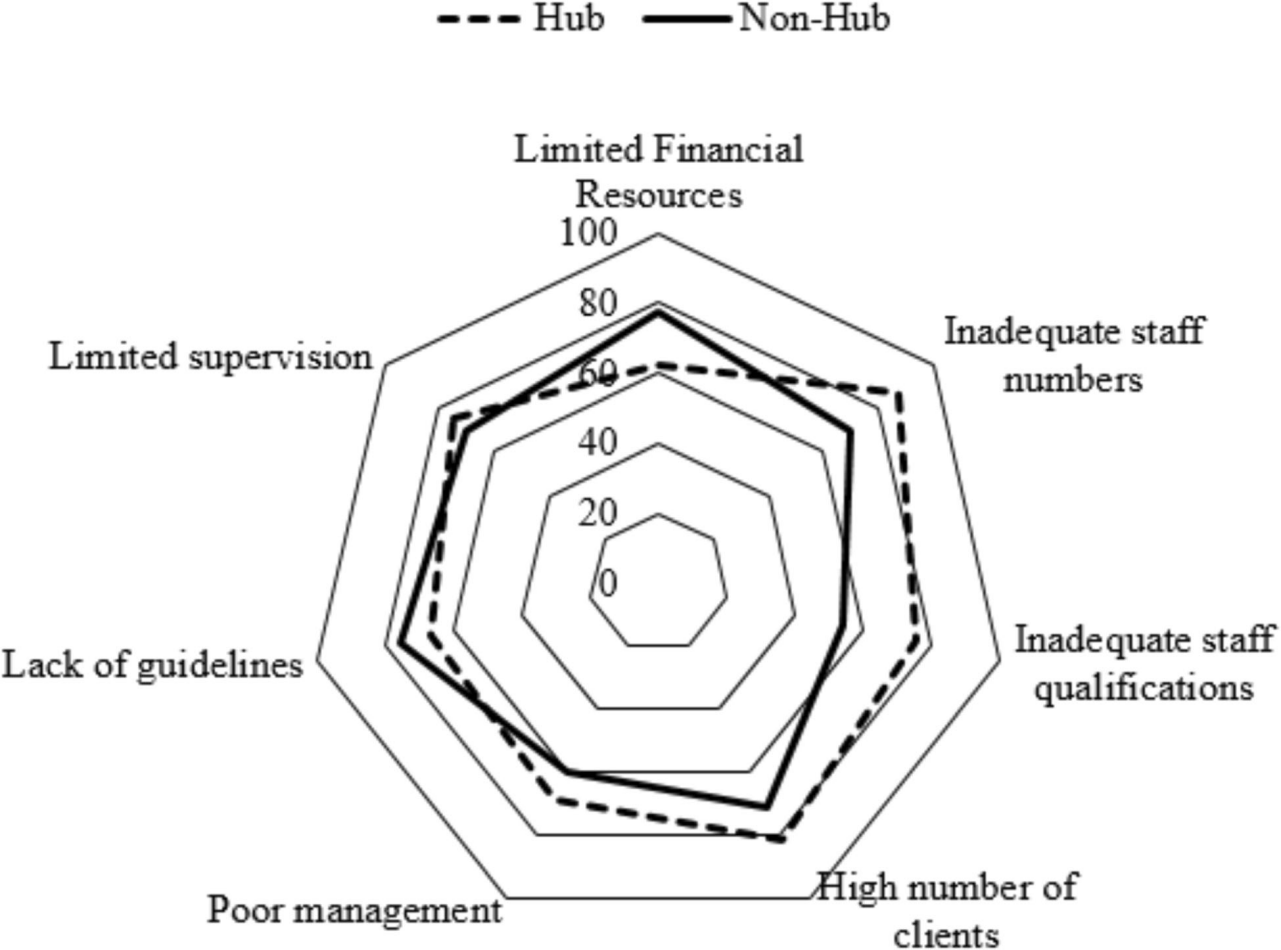
Constraints to human resource strengthening in the laboratory sector for hub and non-hub laboratories in Uganda, 2017

The majority 91/115(79.1%) of the laboratory managers mentioned financial resources, workload and lack of supervision as the major constraints to human resource strengthening in the laboratory sector. Inadequate staff numbers was the main constraint reported in the Northern region affecting almost all 24/25(96.0%) of the laboratories. Lack of guidelines and protocols were the main 23/25 (92.0%) challenges in the Western region. In the West Nile region, inadequate staff qualifications and limited supervision constrained human resources strengthening the most affecting 20/25 (80.0%) of the laboratories. About 14/16(87.5%) of hub laboratories were constrained by inadequate staff numbers, 13/16(81.2%) were constrained by high volume of clients and 12/16(75.0%) were constrained by inadequate staff qualifications (Figs. 2 and 3).

Limited financial resources were also highlighted as one of the most critical constraints to human resource strengthening. It was stated that the lack of finances hinders recruitment of new staff and their motivation, given the high workload. The lack of funds also contributes to poor supervision of the laboratory staffs and low pay.

*“….we have a big problem of remuneration, there is poor pay which affects the attitude of staff and people come here but come for the sake of coming, but the willingness is left home, and I would also say that the work load is much, very much, because technically we are three, one technologist who is the in charge, one lab technician and one lab attendant…”* (KI-Mulago hospital)

*“ok, financially we need pay rise because we get two hundred and fifty thousand shillings per month (USD 68), which is very little. For that reason we need pay rise, then we also need frequent supervision such that if we like some materials we request for them.”* (KI –Maracha Hospital)

## Discussion

Laboratory professionals play a vital role in guiding the course of treatment and the management of diseases, which contribute to improving quality of care. Therefore, strengthening human resources for the laboratory sector ensuring adequate skilled and well-motivated laboratory staffs is critical in the era of GHIs in Uganda.

This study revealed that, generally, laboratories staffing was characterised by an appropriate mix of the cadres ranging from laboratory scientific officers, technologists, technicians, assistants, attendants and microscopists among others with different training backgrounds. This presents an opportunity for a mix of skills, which is crucial in diverse laboratory processes. However, at the different laboratory levels, there was a complete lack of critical cadres for instance; none of the regional referral and national referral hospital laboratories had a microscopist. This puts the whole burden of laboratory workload on the few highly trained cadres. Human resource strengthening initiatives in the laboratory sector therefore need to maintain a delicate balance between recruiting highly trained cadre and lower-level cadres in order to maintain an appropriate skill mix at all levels.

Staffing levels were high with over 90% of the positions filled albeit predominantly by laboratory technicians and assistants. The discrepancies in staffing establishments for instance HCIVs having an excess of laboratory technicians and assistants by 30.0% and 90.0% respectively, with the corresponding lack of other critical cadre, are particularly concerning, indicating unbalanced distribution of laboratory staffs in the sector.

General hospitals and referral hospitals had an excess of over 100% laboratory assistants. This contrasts with previous surveys that reported low staffing levels among various laboratory cadres, with the lowest levels reported at peripheral laboratories [16, 17]. However, the current study revealed a noticeable shortage of highly qualified staff (i.e. laboratory technologists in particular) at general hospitals and regional referral hospitals with only 50.0% and 27.8% filled respectively. This finding is consistent with findings of a survey by Kasusse et al. [16]. The lack of laboratory technologists is most likely explained by the current outdated scheme of service which does not allow recruitment of these cadres. Another explanation would be that most qualified personnel are less attracted to these laboratories due to low payment and the fact that some of these facilities are in remote areas, where the whole country is struggling to attract health workforce [18]. These findings imply that there is need to recruit more highly trained cadres in high-level facilities because these laboratories are designed to handle specialised diagnostics which require specialised skills. Although current staffing establishments at HCIV and HCIII do not allow for laboratory technologists, the available few laboratory staff at these levels had qualifications of laboratory technologists but were working as technicians and some were funded by projects. Clearly, the gaps in the current outdated laboratory staffing policies have driven highly qualified staff to downgrade to a lower qualification in order to get recruited. This finding suggests that there is need to revise the staffing norms to allow the laboratory technologists to be absorbed in these lower health facility-level laboratories. According to Ezeala [19], there is a positive association between highly qualified personnel and quality laboratory services thus stressing the need for highly qualified personnel with laboratory skills to manage laboratories in Uganda.

The training of laboratory staff is key to the successful operation of laboratories. In this study, however, onsite training for laboratory personnel was low (43.9%) especially within lower-level health facility laboratories and this was uniformly low across the four regions (Eastern, Western, West Nile and Northern region). According to the National Health Laboratory Strategic Plan (2010-2015), most of these trainings have not been responsive to individual needs [17]. All laboratory personnel must receive direct and detailed job-specific training and continuing education to perform all duties so that they understand and competently carry out the necessary functions [20]. These study findings underscore the need to conduct regular trainings for laboratory staffs in an attempt to strengthen human resource for the laboratory sector in the era of GHI in Uganda.

Almost all the laboratories had ever been supervised and more than half were supervised within the month preceding the survey, with the supervisions focusing on multiple programmes mainly TB and HIV activities. This provides an opportunity to build the capacity of staff to offer quality services. A Northern Uganda Malaria AIDS Tuberculosis Program (NUMAT) technical brief 2011 had previously reported an improvement in the laboratory supervision especially HC III facilities from 61% in July 2008 to 75% in May 2011. However, this was contrary to what was reported in Uganda National Health laboratory policy [12] where very few laboratories got quarterly technical support supervision due to limited capacity of the supervisory bodies. The policy further revealed that only facilities involved in vertical programmes such as those related to TB and HIV/AIDS do get some regular in-service training and supervision [12]. These findings highlight the need to integrate supervision and make it more comprehensive at each visit instead of focusing on only a few aspects.

Financial resources and workload were the major constraints to human resource strengthening in the laboratory sector across the different laboratories in the different regions. This finding is consistent with the Uganda National Health Laboratory Services Policy (2009), which revealed that health laboratories were underfunded and had limited staff. The lack of financial resources could be attributed to the absence of a dedicated budget line for laboratory services [12]. Financial resources are critical to strengthening human resource since they facilitate the different human resource strengthening initiatives including recruitment of staffs, staff development activities and motivation. Therefore, this finding implies that there is need for an ample dedicated budget for the laboratory sector to cater for human resource strengthening activities

Furthermore, inadequate staff numbers and workload were reported to be the main constraints in almost all the laboratories in the Northern region. These study findings imply that work-based staffing models need to be revised to ensure adequate staff numbers and skill mix. Additionally, strategies need to be developed to recruit adequate numbers of laboratory technologists and scientists especially at high-level laboratories.

### Study limitations

The staffing norms used are based on the level of facility instead of being workload based. This may not really give a true estimate of the workload-staffing gap. However, our results give insights on the human resource gaps per health facility level in the country representative of the four regions of the country.

## Conclusion

The laboratory sector in Uganda is constrained with inadequate staffing especially the high-level cadres with and excess of lower-level cadre. Inadequate skill mix and escalating workload further constrain service quality. Training has been mainly done for staff under donor-funded programmes like HIV, TB and malaria. The Ministry of Health needs to develop work-based staffing models to ensure adequate staff numbers and skill mix and also to ensure that laboratory technologists and scientists are accommodated especially at high-level laboratories. Training needs to extend beyond HIV, TB and malaria.

## Data Availability

The datasets used and/or analysed during the study are available from the corresponding author on reasonable request.
